# Autoantibodies in neuromuscular transmission disorders

**DOI:** 10.4103/0972-2327.42932

**Published:** 2008

**Authors:** Angela Vincent

**Affiliations:** Department of Clinical Neurology and Weatherall Institute of Molecular Medicine, University of Oxford, UK

**Keywords:** Acetylcholine receptor antibody, myasthenia, neuromuscular junction

## Abstract

It is a great pleasure to be asked to honour the memory of Dr. Baldev Singh by reviewing the field of autoantibodies in myasthenia gravis and other neurotransmission disorders. The neuromuscular junction (NMJ) is the site of a number of different autoimmune and genetic disorders, and it is also the target of many neurotoxins from venomous snakes, spiders, scorpions and other species. The molecular organization of the NMJ is graphically represented in Figure 1A, where different ion channels, receptors and other proteins are shown. Four of the ion channels or receptors are directly involved in autoimmune diseases. This brief review will not only concentrate on these conditions but also illustrate how their study is helping us to understand the etiology of rare but treatable neurological syndromes of the central nervous system.

## Myasthenia gravis

In myasthenia gravis (MG), the target of the antibodies is the acetylcholine receptor (AChR), and the antibodies are measured by immune precipitation of AChRs. These are solubilised from the human muscle or from muscle cell lines and then radiolabelled with ^125^I-alpha bungarotoxin. Alpha-bungarotoxin is an 8000 MW polypeptide from the venom of Bungarus multicintus, the Taiwan banded krait, that binds specifically and irreversibly to the AChRs. The structure and immunology of the AChR has been reviewed by Tzartos *et al.* 1998.[1]

**Figure 1:**
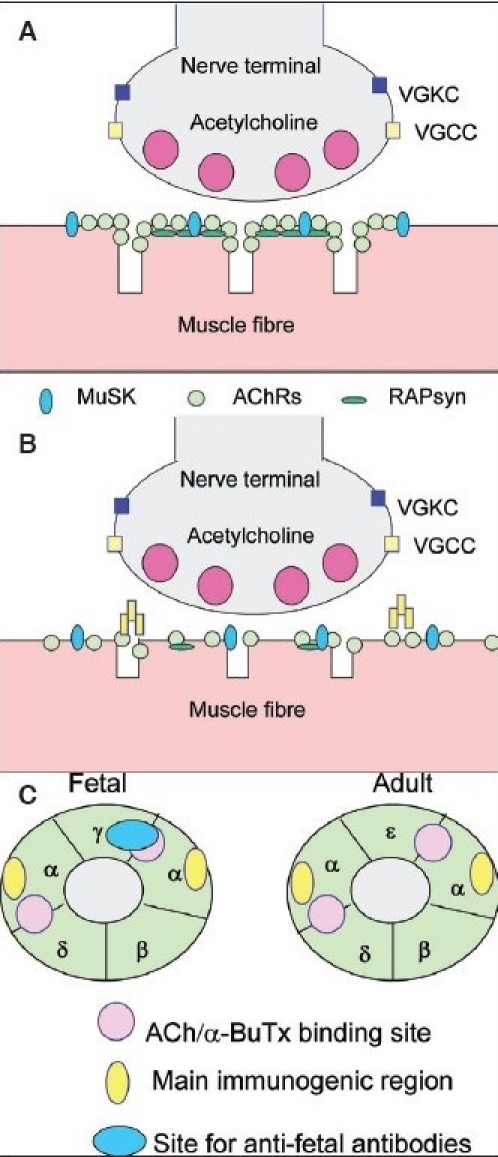
(A) The neuromuscular junction showing the targets for antibodies in disease. Rapsyn is the cytoskeletal protein that clusters the acetylcholine receptors. (B) The neuromuscular junction in myasthenia gravis with AChR antibodies. The AChRs are reduced in number and there is morphological damage to the postsynaptic membrane. (C) The AChR viewed from above the membrane consists of five subunits, two alphas, one beta, one delta and either a gamma (fetal type) or epsilon (adult type). Bungarotoxin and acetylcholine bind to sites on the interfaces between the alpha subunit and adjacent subunits. Many, but not all, antibodies bind to a region known as the main immunogenic region on the alpha subunits. Mothers with babies who are born with arthrogryposis may have antibodies that bind to a gamma-subunit specific site

The main defect in MG is loss of the AChRs on the postsynaptic membrane [Figure 1B]. The importance of the AChR antibodies in causing myasthenia was demonstrated principally by two simple experiments. Firstly, passive transfer of immunoglobulins from patients with MG to mice was associated with clinical and electrophysiological evidence of MG in the mice.[2] Secondly, plasma exchange was found to be highly effective in MG, even in patients who had been bed-bound for many years, and the patients' clinical symptoms mirrored the AChR antibody levels during and after the treatment.[3] Much of the history of myasthenia research before and after this time is reviewed briefly elsewhere.[4,5]

We now know that the AChR is a pentameric membrane protein consisting of two alpha, one beta, one delta and one epsilon subunit in the adult muscle, whereas, during development, the gamma subunit takes the place of the epsilon [Figure 1C]. The AChR antibodies are principally IgG1 subclass and bind to the extracellular domain of the AChR and cause loss of functional receptors by a combination of complement-mediated damage, antibody-mediated down-regulation and direct pharmacological block.[4,6]

Myasthenia gravis patients can be divided into several subtypes. The most clearly defined are early-onset MG, late-onset MG and thymoma-associated MG. In addition, there are patients with MuSK antibodies (see below). Those patients who are negative for both AChR and MuSK antibodies are called “seronegative” (SNMG).[7] The patients in these subgroups are partially differentiated by their male to female ratios, HLA associations and thymic pathology [Table 1]. Interestingly, it is becoming increasingly clear, at least in Western populations, that MG is more common in older people than thought previously.[8] The late-onset group tends to have an atrophic thymus and to be associated with HLAB7 DR2.

**Table 1:** Main types of myasthenia gravis

Type	Thymus	HLA	AChR Ab
Early onset(<40 years)	Hyperplastic	B8DR3	AChR
Late onset(>40 years)	Atrophic	B7DR2	AChR
Thymoma	Tumour	None	AChR
MuSK-MG	Normal	DR5	MuSK
“SNMG”	Hyperplastic	?	AChR*

* AChR antibodies only detected by binding to clustered AChR, see Antibodies in SNMG

## Maternal antibodies and MG

Antibodies can cross the placenta in large amounts, from around week 16 in gestation, and the phenomenon of transient neonatal MG is well established, although relatively few cases are seen nowadays, perhaps because of better treatment of the mothers. Very rarely, babies are born with more permanent damage that includes arthrogryposis multiplex congenital. Lung hypoplasia can lead to neonatal death. A small number of MG patients who have had recurrent pregnancies affected by this syndrome have been shown to have high levels of antibodies that bind specifically to the fetal form of the AChR and block its function [Figure 1B]. Thus these antibodies cross the placenta and paralyze the baby during development, leading to fixed joint contractures and other deformities.[9] A small number of women without evidence of MG also have these antibodies, which can lead to fetal damage. Since most laboratories use a commercial mixture of adult and fetal AChR to test for antibodies in MG, it should be relatively simple to test the maternal sera to see if they have antibodies to AChR even when the mother is asymptomatic.

## Thymoma-associated and late-onset MG

Thymomas are found in around 10% of MG patients and can be associated not only with MG but with a range of neurological and haematological disorders; they often have antibodies to neuronal and muscle antigens, and to certain cytokines. Antibodies to striated muscle proteins are not measured routinely in many laboratories because the presence of a thymoma can usually be seen on CT scans. Moreover, striated muscle antibodies, now shown to target the muscle proteins titin and ryanodine receptor,[10] are also found in late-onset MG and in thymoma-associated MG [Table 1]. The role of the thymoma in inducing these antibodies is still not clear; it is likely that the thymic tumour generates many T cells that induce autoimmunity in the periphery, but it is possible that some of the autoimmunity occurs in the surrounding normal tissue.[11]

## Early-onset MG

The role of thymus in early-onset MG is much clearer. It has been known for many years that some of the AChR antibody is made in the thymus, and thymectomy in early-onset MG can lead to clinical improvement and a fall in AChR antibodies, although other treatments are also required in most cases. Evidence of AChR expression on thymic muscle-like myoid cells explains, to some extent, the appearance of the germinal centres and the AChR antibody synthesis. In fact, recent studies demonstrate not only the presence of the myoid cells around and within the germinal centres, but also complement components deposited on the thymic epithelial and myoid cells.[12] Interestingly, the myoid cells do not express the complement regulators, CD55 and CD59, suggesting that they might be particularly vulnerable to attack by AChR antibodies. Thus it is highly likely that the immune response in early-onset MG is partly induced in the thymus, and/or that it is maintained there by the presence of AChRs on the myoid cells.[12]

## MuSK-MG

A proportion of the patients with typical generalized MG do not have AChR antibodies [Table 1]. A variable number of these patients will, instead, have antibodies to muscle specific kinase (MuSK).[13] MuSK is a membrane receptor tyrosine kinase that is highly concentrated at the NMJ in mature muscle. The antibodies are measured by immunoprecipitation of MuSK that has been radioactively labelled.[14] Interestingly, MuSK antibodies are not found equally within the MG population worldwide, and their prevalence appears to correlate with latitude, being found most frequently in the northern hemisphere in MG patients who live within latitudes 30 and 50 N of the equator. (A Vincent unpublished results). There is insufficient data from India, so far, to say whether this geographical distribution is evident within the subcontinent.

MuSK MG is often relatively severe, with high grades at presentation and at maximal severity.[15,16] [Table 2]. Many patients respond to immunosuppressive treatments with a fall in severity grade, but they often require more treatment, such as cyclosporine A, in order to achieve a remission compared with patients who have AChR antibodies or with those who have neither AChR nor MuSK antibodies.[17]

**Table 2:** Clinical features and treatment responses in MuSK-MG and SN-MG patients

	MuSK-MG	SN-MG
MGFA grade at presentation	Moderate	Low
MGFA grade at max severity	Severe	Moderate
MGFA final grade	Low	Low
Additional therapies used	Common	Uncommon
Eg. Cyclosporine A		

Data from the International SNMG Survey (unpublished)

One notable feature of MuSK-MG is the frequent involvement of the facial and bulbar muscles, which often remain weak even when the other muscles have recovered. Indeed, neuromuscular transmission is compromised in these muscles even when limb muscles show normal function,[18] and in some patients the facial and tongue muscles are reduced in volume, as shown by MRI[19] with high signal in the atrophic tongue muscle. These findings correlated with the duration of steroid treatment, but it is not clear whether the myopathy reflected the duration of severe disease or was related to the duration and extent of steroid therapy.

## The thymus in MuSK-MG and SNMG

It was important to look at the pathology of the thymus in patients with MuSK antibodies, and to compare it with both typical AChR-MG and with those patients with neither antibody (SNMG). Two studies[20,21] showed that the MuSK-MG thymus is normal or only very slightly affected. It seldom shows germinal centres and those that are present are small and similar to the small germinal centres that can be found in healthy thymus. There is also little or no activation of complement in the thymus.[12] Thus these observations suggest that the thymus in MuSK-MG patients does not show any of the changes found in AChR-MG patients, and MuSK-MG would not necessarily benefit from thymectomy. Although there is no objective evidence regarding thymectomy in MuSK patients, some centres do not offer it.

By comparison, thymus from those patients with neither antibody (seronegative MG, SNMG) often shows evidence of abnormal immune activity. Germinal centres and lymphocytic infiltrates are present, in many cases comparable to those in typical AChR-MG patients. There is also evidence of complement deposition on thymic epithelium and myoid cells.[12]

## Antibodies in SNMG

A number of previous studies from our laboratory showed that SNMG sera or plasmas could inhibit the function of AChRs expressed on cultured cell lines. The effect was evident within a few minutes and was reversible with washing.[22,23] Together with the finding that SNMG thymus is very similar to that of AChR-MG patients, we hypothesized that the SNMG patients possessed an AChR antibody that did not bind to the solubilised AChR that is used in routine diagnostic testing, but might bind to the AchR on the muscle cell surface. However, we could not detect antibody binding by immunofluorescence using AChR-expressing cell lines, perhaps because the antibody was rapidly washed off.

**Figure 2:**
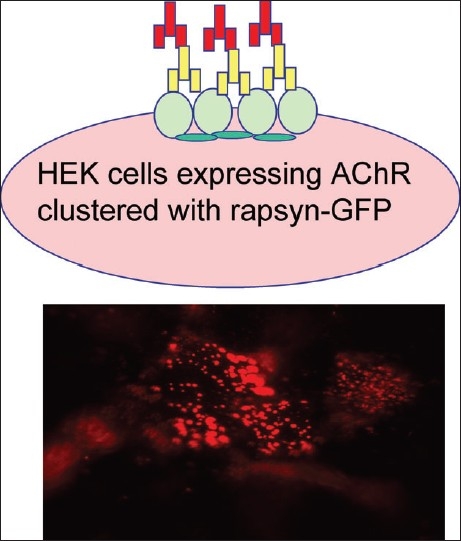
Binding of “low avidity” antibodies to AChRs clustered by rapsyn. For this, human embryonic kidney cells were transfected with DNA to induce expression of the AChR subunits and rapsyn, and antibody binding can be detected by immunofluorescently-labelled anti-human IgG, as diagrammatically illustrated on the left and shown on the right. The AChRs are highly clustered into patches on the surface of the cells. Patients previously negative for antibodies binding to AChR in solution, often show evidence of antibodies that bind to clustered AChR as illustrated for one serum

At the neuromuscular junction, the AChRs are present at very high density. This is due to clustering by the cytoskeletal protein RAPsyn [Figure 1A]. When we introduced the DNA for RAPsyn into the AChR-expressing cells, the AChRs redistributed into high density clusters [Figure 2]. Many of the SNMG sera could then be shown to bind. It is likely that this is because the high density of the AChRs in the clusters allows relatively low avidity AChR antibodies to bind divalently to adjacent receptors,[24] although this has not yet been demonstrated unequivocally.

## The Lambert Eaton myasthenic syndrome

The Lambert Eaton myasthenic syndrome (LEMS) is a well established antibody-mediated disease in which antibodies to voltage-gated calcium channels (VGCC) cause loss of calcium channel function at the neuromuscular junction.[25] As a result, the release of transmitter is compromised and the patient develops a typical pattern of weakness. During repetitive stimulation at high frequency, or following sustained voluntary contraction, the muscle strength improves, probably because during repeated motor nerve activity, calcium accumulates within the nerve terminal and neurotransmitter release increases. Reflexes are often weak or absent. Plasma exchange and intravenous immunoglobulins are both effective treatments,[26] but in many cases it is sufficient to give steroids and azathioprine to reduce the immune response, and 3,4 diamino-pyridine, which will increase the ACh release.

LEMS is a paradigm for paraneoplastic disorders. In around half of the patients, a small cell lung cancer (SCLC), or less commonly another tumour, is present. SCLCs express calcium channels.[27] It is thought that the antibodies in these patients are primarily directed at the tumour cells and cross-react secondarily with their targets at the motor nerve terminal. VGCC antibodies are found in 85% of LEMS patients and are highly specific, but they can also be found in some patients with SCLC and cerebellar ataxia, without obvious LEMS.[28] The antibodies bind specifically to the P/Q-type VGCCs that are found throughout the nervous system, mainly at synaptic terminals. These VGCC are specific targets for a cone snail toxin, w-conotoxin MVIIC, and the antibodies are measured by radioimmunoprecipitation of ^125^I-w-conotoxin-VGCCs.[29]

Interestingly, the antibodies in LEMS do not cause complement-mediated damage to the motor nerve terminal, perhaps because it is well protected by complement regulators. The antibodies cross-link the VGCCs in the membrane and lead to their redistribution and internalization (reviewed in).[30]

## Acquired neuromyotonia and other VGKC-antibody related conditions

Acquired neuromyotonia is a heterogeneous condition, but at least in many cases is caused by autoantibodies. Patients complain of muscle twitching, stiffness, cramps and often excessive sweating. Sensory symptoms are often common.[31] The condition is due to hyperactivity of the motor nerves and the hyperactivity is thought to be generated mainly in the distal portion of the nerves or even at the motor nerve terminal. Antibodies to voltage-gated potassium channels (VGKCs) are found in about 40% of these patients, although they are more common in the 20% who have thymomas. The patients respond to plasma exchange,[32] but neuromyotonia is not life-threatening and many patients do well on anti-epileptic medication alone and do not require immunological treatments.

The antibodies are measured by radioimmunoprecipitation of VGKCs that have been labelled with ^125^I-dendrotoxin, a mamba toxin.[33] Since this toxin binds to three different VGKC subtypes, Kv1.1, 1.2 and 1.6, it is not altogether clear which channel is the target for the antibodies; experiments are in process to try to determine the target more precisely.

Some patients with neuromyotonia also have central symptoms. A condition known as Morvan's syndrome includes neuromyotonia, autonomic dysfunction with cognitive and sleep disturbance. Although very rare, a number of patients have now been identified and shown to have high levels of VGKC antibodies and to respond very well to immunotherapies.[34,35]

Finally, VGKC antibodies are now being found with increasing frequency (approximately one per week in the UK, compared to about 20 new cases of MG diagnosed per week) in patients with a form of limbic encephalitis[36,37] and in related conditions. These patients usually show high signal in the hippocampi/medial temporal lobes and often present with low plasma sodium levels. They usually have significant memory loss and develop seizures, either localized or general, which can be resistant to anti-epileptic medication. VGKC antibodies can be very high and fall rapidly after immunotherapies, associated with resolution of symptoms. The condition seems to be monophasic in most patients since the antibodies do not rebound when treatments are stopped. Interestingly, these patients also may have sleep disturbance and the spectrum of clinical syndromes associated with VGKC antibodies is widening with greater recognition.[38]

**Figure 3:**
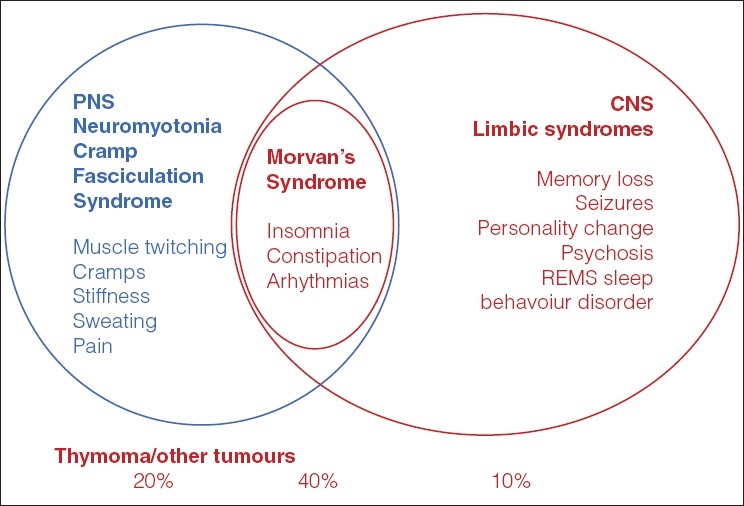
Disorders associated with potassium channel antibodies. These antibodies were originally detected in patients with neuromyotonia and cramp fasciculation syndrome. They were then found in patients with Morvan's syndrome which includes autonomic and CNS involvement. Subsequently patients without peripheral symptoms were found to be positive. Thymomas are found in each of these syndromes, but the incidence appears to be variable

It is important to remember that the patients can have thymomas[39] or other tumours,[38] although in our experience the majority of patients with high VGKC antibodies have a non-paraneoplastic condition. Figure 3 illustrates the different VGKC antibody-associated syndromes and their tumour frequencies. VGKC antibodies are also being found in a small number of patients with epilepsy, without obvious cognitive disorders.[40,41,42] In some of these, the levels of VGKC antibodies are not very high and the antibodies could be an epiphenomenon. In others, the impressive response to immunotherapies strongly suggests that the antibodies are pathogenic.[40,42]

## Final comments

The field of antibody-mediated diseases has gone far since the first evidence of antibodies to AChRs in the 1970s. There are now four distinct diseases of the neuromuscular junction, caused by antibodies to different targets, and a growing number of CNS conditions that are likely to be caused by similar mechanisms. These include nonparaneoplastic and paraneoplastic forms of autoimmune encephalitis with antibodies to VGKCs (see above), NMDA receptors[43] and glycine receptors,[44] and to the water channel aquaporin-4 in neuromyelitis optica.[45] The new techniques for their detection, such as cell-based approaches [Figure 2] hold great promise for identification of new antibodies and for improved diagnosis, thus better treatment for the patients.
